# Protocols.io: Virtual Communities for Protocol Development and Discussion

**DOI:** 10.1371/journal.pbio.1002538

**Published:** 2016-08-22

**Authors:** Leonid Teytelman, Alexei Stoliartchouk, Lori Kindler, Bonnie L. Hurwitz

**Affiliations:** 1 protocols.io, Berkeley, California, United States of America; 2 Department of Agricultural and Biosystems Engineering, University of Arizona, Tucson, Arizona, United States of America

## Abstract

The detailed know-how to implement research protocols frequently remains restricted to the research group that developed the method or technology. This knowledge often exists at a level that is too detailed for inclusion in the methods section of scientific articles. Consequently, methods are not easily reproduced, leading to a loss of time and effort by other researchers. The challenge is to develop a method-centered collaborative platform to connect with fellow researchers and discover state-of-the-art knowledge. Protocols.io is an open-access platform for detailing, sharing, and discussing molecular and computational protocols that can be useful before, during, and after publication of research results.

## Abstract 2

This Community Page presents an open-access platform, protocols.io (https://www.protocols.io/), which enables collaborative sharing and discovery of state-of-the-art research methods.

## Introduction: The Changing Landscape of Science Publishing

The act of publishing research serves to communicate discoveries and results. However, to truly advance knowledge and scientific understanding, scientists are expected to share material, methods, and data so that others may reproduce and build upon the published work. When publishing scientific articles, it is standard practice for authors to deposit DNA and protein sequences into public repositories such as GenBank, EMBL, or DDBJ. Over the past decade, journals have extended these open science practices to include all available associated data, statistical methods, and code. Yet, there is a growing recognition that disparate data provided by individual authors upon request—on their websites or in supplementary information files—is far less desirable than pooling resources in curated databases. Hence, many publishers now guide authors to share supplementary data files via DataDryad, figshare, or similar repositories and make software available in code repositories such as GitHub. The PLOS Data Sharing policy [1] is a good example of this paradigm shift. Yet, simply sharing data and code is not enough to allow for scientific reproducibility, resulting in a rising focus on the importance of reporting detailed methods. Journals such as *Nature Protocols* [2], *JOVE* [3], and *MethodsX* [4] have begun to fill this void in the last decade. Moreover, attempts have been made to create central open-access communities where protocols can be crowdsourced, including BioProtocols [5], Open Wet Ware [6], and Nature Protocol Exchange.

In creating protocols.io, we extended the idea of an open, up-to-date protocol repository, applying lessons learned from previous efforts. Our goal was to create a platform that serves researchers’ reporting of methods, with benefits before, during, and after publication of findings, in the same way GitHub serves software developers. Here, we describe the development of the protocol.io platform as a resource for researchers to share and discover experimental and computational methods.

## Integration of Protocols.io into the Research Process

**Fig 1:**
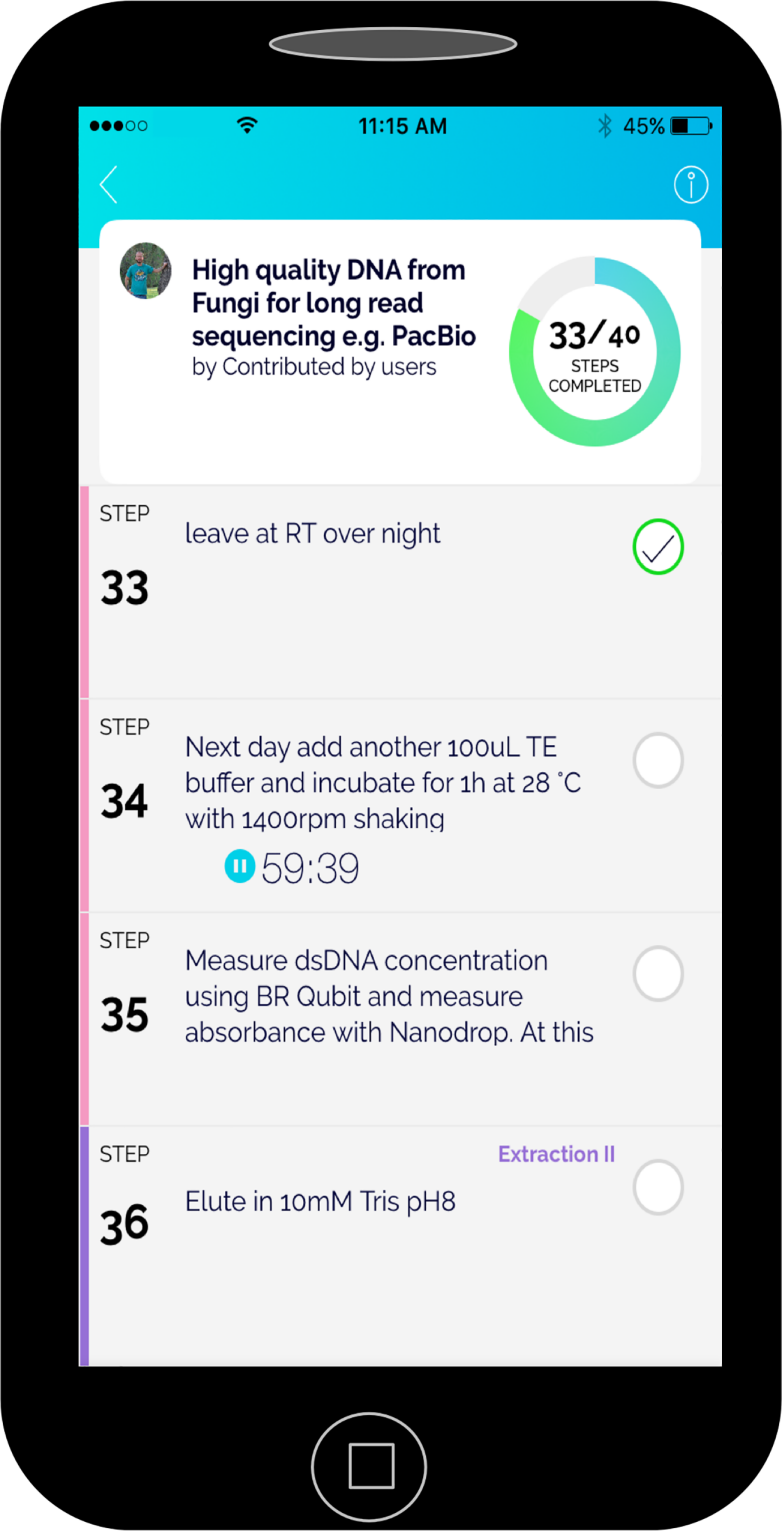
Running a protocol in protocols.io using a mobile device. Users can run protocols in protocols.io and annotate any departures from the existing protocol for later reference in lab notebook–like records.

Protocols.io can be used at any point in the research cycle, long before the results or methods are ready for public sharing. Every new protocol is private by default to the user who created it. In contrast to a PDF or paper copy, each protocol may be followed step-by-step during experimental lab work. That is, the protocols are “runnable” on the web and on the native iOS and Android apps, in a checklist manner. At run time, the user can note changes to individual steps, as performed at that moment (Fig 1). These features help researchers keep track of the position in the experiment and create a precise electronic record of the experimental detail and changes to the implementation of a protocol. These lab notebook–like records reduce the need for handwritten notes and can be exported in a variety of formats.

Personal protocols can also be shared with individuals and groups for collaborative development. Similarly to Google Docs, sharing permissions can be specified to allow editing or viewing only. Moreover, individual researchers can form groups that can be public or private, helping to organize commonly used methods and facilitate discussion among colleagues and collaborators.

The platform can be used privately or collaboratively during research to facilitate reproducibility by capturing methodological nuances in a given field or for a particular software suite. Specifically, users can add detailed annotations on protocols attached to specific steps, including example datasets, reagents or equipment, links to code repositories, cyberinfrastructure platforms [7,8], or virtual machines. Moreover, steps in protocols can include images and videos to better communicate the visual intricacies of experimental or computational techniques.

## Use of Protocols.io during Manuscript Submission

In light of concerns over the reproducibility of published research, the journals *Genetics* and *GigaScience* have partnered with protocols.io and are encouraging authors to share detailed methods via protocols.io instead of supplementary files. A private reviewer link is available when submitting work for publication so that reviewers can access protocols associated with the paper without having to create an account on protocols.io.

Early adopters have also begun to include the links to protocols.io in their submissions and publications, independent of formal integration of the platform with journals. The following are examples of recently published papers that cite protocols.io, where DOIs can be reserved via CrossRef prior to formal publication for a journal-friendly citation to protocols.io from a paper’s materials and methods section [9,10].

## Postpublication: Keeping the Methods Current

After the publication of a research paper, the underlying protocols are frequently optimized by the authors or other researchers using the methods; mistakes are discovered, steps are modified for new applications, and details are optimized to improve the protocol or to take advantage of a new kit or reagent. However, there is no central infrastructure that enables scientists to share corrections and improvements of methods that have been already published. Protocols.io was created explicitly to address this gap.

Once published, a protocol cannot be edited or removed, but authors can easily create new versions. This preserves the scientific record and ensures that DOI links to protocols.io from publications do not break. At the same time, a “master” version of any protocol can be maintained, and visitors who land on earlier versions can be alerted that an updated version exists [11].

While versioning is available strictly for the author, anyone can fork (copy) a public method, modify it, and publish their changes as a new derivative with a clear link to the original. Furthermore, step-level and protocol-level comments enable discussions. The author is automatically notified of all questions and annotations, allowing the author or other researchers to respond publicly. Thus, instead of repeatedly answering the same questions by email, scientists can make their responses public. This functionality allows for an FAQ-like enrichment of the published protocols, with hints, clarifications, and updates accumulating over time (see example [12]).

## Virtual Communities

To enhance virtual collaboration and sharing, groups are not limited to individual labs or companies; broader virtual communities or “super-groups” can be created around a field or topic, such as the VERVE Net group for viral ecologists [13,14].

Virtual communities are method-centered but have a number of additional features that allow researchers to share up-to-date information, news, jobs, conferences, and events with their virtual communities. Groups can also maintain a shared literature reference library specific to their area of interest and get recommendations of newly published papers in PubMed.

The key mission of this group functionality is to encourage community collaboration and to make it easier for new researchers joining a given field to get help and to discover state of the art methods and key papers. Moreover, authors can highlight collections of protocols associated with a paper they recently published.

## Protocols.io Is Open Access

Protocols.io is open access—it is both free to read and free to publish. Every protocol can be exported as PDF or json, and public APIs are available [15]. All accounts are free, there is no charge for the mobile apps, and all public protocols can be viewed without registration or sign-in.

As a private company in the science communication sphere, protocols.io recognizes the need to ensure digital preservation of the public protocol knowledge. In addition to the export and public APIs mentioned above, protocols.io partners with the Center for Open Science [16] to mirror all public content. Protocols.io is also developing archiving with CLOCKSS [17], as is common for established research journals.

Protocols.io relies on subscription revenue to its analytics dashboard, allowing vendors and publishers to study in real time the adoption and experimental use of their protocols and reagents. Private information about the users and email addresses are never shared. All data in analytics are anonymized and aggregated; modifications are never shared unless public.

**Fig 2:**
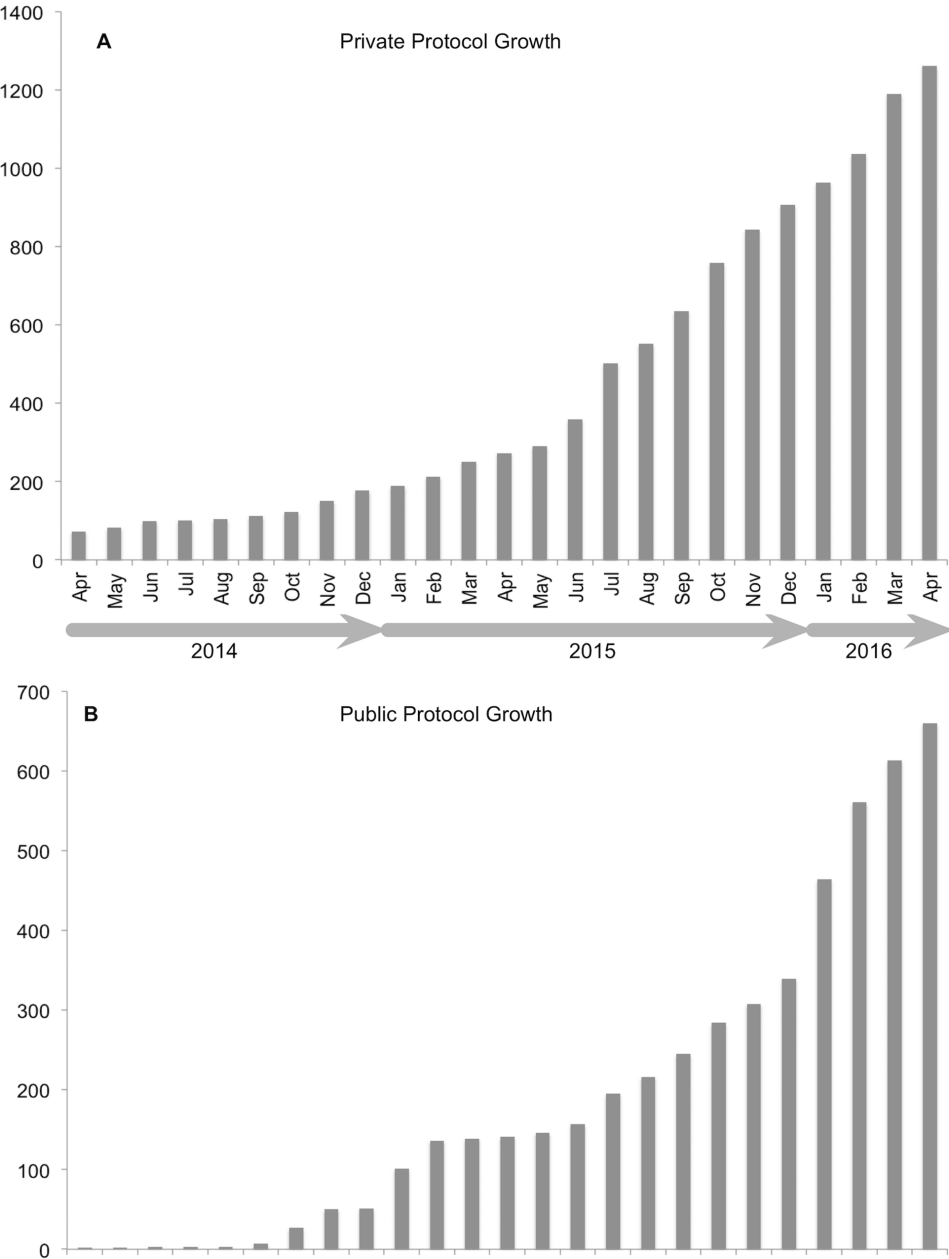
Growth in public and private protocols in protocols.io from April 2014–2016.

While protocols.io partners with vendors and publishers, the core mission is to serve as a crowdsourced community resource for both academic and industry researchers. The steady growth of the methods on protocols.io is mostly driven by academic scientists around the world, with over 2,000 protocols developed since April 2014, of which 800 are public (Fig 2).

## Abbreviations

API: Application Program Interface
CLOCKSS: Controlled Lots of Copies Keep Stuff Safe
VERVE Net: The Viral Ecology Research and Virtual Exchange Network
